# Continuous monitoring of brain perfusion by cerebral oximetry after spontaneous return of circulation in cardiac arrest: a case report

**DOI:** 10.1186/s12883-022-02880-2

**Published:** 2022-09-22

**Authors:** Heng Zhou, Caiwei Lin, Jiawei Liu, Xudong Wang

**Affiliations:** Department of Emergency Medicine, Aerospace Center Hospital, 15 Yuquan Road, Haidian District, 100049 Beijing, China

**Keywords:** Cerebral oximetry, Return of spontaneous circulation, Cardiac arrest, Intra-aortic balloon pump, Case report

## Abstract

**Background:**

Cerebral resuscitation determines the prognosis for patients who have experienced sudden death, and brain protection is the focus of clinical treatment. Cerebral resuscitation depends on the timing and quality of cardiopulmonary resuscitation (CPR). At present, cerebral oxygen monitoring is used mainly to monitor the quality of external cardiac compression and provide a prognosis for the nervous system. However, after the return of autonomous circulation, it is necessary to conduct continuous monitoring to ensure measures are taken timeously since hemodynamic instability, brain edema, and other factors may cause occult brain injury, and invasive arterial pressure cannot represent cerebral perfusion.

**Case presentation:**

By using continuous cerebral oxygen monitoring after CPR and the return of spontaneous circulation, a patient who was witnessed to have experienced sudden death in the hospital was found to have insufficient cerebral perfusion; he underwent timely intra-aortic balloon counterpulsation to improve his hemodynamics and cerebral perfusion. The patient went on to achieve a good neurological prognosis.

**Conclusion:**

Cerebral oxygen monitoring should be conducted throughout the treatment period; physicians should understand cerebral perfusion in real time and implement timely intervention measures to reduce occult brain injury and improve the neurological prognosis of patients.

## Background

After successful cardiopulmonary resuscitation (CPR), the overall prognosis of patients who have experienced sudden death is poor. The success rate of CPR for out-of-hospital sudden death is 7.6% in Europe, 6.8% in North America, 3.0% in Asia, and 9.7% in Australia [1]. The main reasons for this low success rate are that CPR for out-of-hospital sudden death is not conducted timeously, and the quality of the CPR is not of a high standard. However, the success rate of CPR for in-hospital sudden death is also low, with some large studies reporting a success rate of approximately 20% [2]. The reasons are as follows: After successful CPR, occult cerebral hypoperfusion persists as a result of factors such as cardiogenic sudden death, myocardial infarction complications, and circulatory instability. Often, this hypoperfusion is not identified, and timely measures are not implemented to correct it. At present, most monitoring methods have a limited effect and cannot reflect cerebral perfusion accurately in real time. In current studies, cerebral oxygen monitoring is mainly used to guide CPR [3]. The present case study revealed that routine cerebral oxygen monitoring after successful CPR may help identify problems, guide treatment, and further improve patients’ neurological prognoses.

## Case presentation

Herein, we report a case in which cerebral oximetry was used to optimize brain perfusion in a patient with a return of spontaneous circulation (ROSC) after cardiac arrest (CA). A 67-year-old man (height = 172 cm, weight = 55 kg) suffered a CA secondary to acute myocardial infarction. His past medical history included hypertension, diabetes mellitus, and chronic renal failure (stage 5). The patient had a 15-min history of chest distress and sweating, and CA occurred as the ambulance arrived at the emergency department. Mechanical CPR was started using the LUCAS 2 Chest Compression System (Medtronic, Minneapolis, USA), an intravenous infusion of various drugs was administered, and monitoring of frontal oxygenation using near-infrared spectrophotometry (INVOS^™^ 5100 C Cerebral/Somatic Oximeter, Medtronic) was started. An ROSC occurred 8 min later, and an electrocardiogram indicated extensive ST-segment elevation in leads V2–V5. The regional cerebral oxygen saturation (rSO2) levels were 70–80%. Cardiologists performed a coronary artery balloon dilation and administered 300-mg aspirin, 180-mg ticagrelor, and 5,500-IU heparin within 3 h of admission. However, the patient’s pupils became dilated, and they reacted slowly to light. His consciousness deteriorated. The rSO2 showed a continuous decline from 80 to 21%, with little change in the arterial blood gas analysis, but the heart rate, blood pressure, and ejection fraction (EF) measured by ultrasound cardiogram decreased from 130 to 90 bpm, 180/90 to 99/58 mmHg, and 46–33%, respectively. A left ventricular aneurysm was observed. An intra-aortic balloon pump (IABP) was fitted to maintain the patient’s blood pressure at 130–140/70–90 mmHg.

The next day, the patient felt mild–moderate pain, their EF was 55%, and their rSO2 increased gradually from 21 to 50%. Five days after PCI passed away, the IABP was removed successfully, the patient’s vital signs were stable, most indicators were improving, and the patient’s neurological prognosis was good (cerebral performance category = 2).

## Discussion

At present, the static value of brain oxygenation as an indicator of neurological prognosis is an important research topic [4].

In the past, cerebral resuscitation treatment has primarily consisted of providing treatment and observing the outcome without conducting any monitoring. At present, brain oxygen monitoring is not implemented routinely. A mean arterial pressure of > 65 mmHg does not represent good cerebral perfusion [5]. In this case, through the continuous monitoring of brain oxygen levels (in which changes in values were monitored to identify and explain problems that had occurred), a possible brain function injury caused by a hemodynamic disorder was revealed; timely measures were implemented to avoid further brain injury, and a good neurological outcome was achieved. This type of monitoring can further improve a patient’s overall neurological prognosis. Therefore, routine cerebral oxygen monitoring is recommended. However, further studies should be conducted on patients who experience sudden death to compare the neurological prognoses between those who receive cerebral oxygen monitoring and those who do not.

Currently, brain damage is the most important cause of morbidity and mortality in survivors of CA, and an rSO2 level of < 25% increases the likelihood of death and poor neurological outcome [6–8]. Despite adequate resuscitation, occult cerebral ischemia can still occur. Therefore, it is necessary to conduct cerebral oxygen monitoring in real time. Several studies have shown that the use of near-infrared spectrophotometry (cerebral oximetry) is feasible since it reliably reflects perfusion [9] and offers real-time noninvasive monitoring [10]. Recent evidence suggests that the use of cerebral oximetry could illustrate the quality of resuscitation during CA [11]. In this case study, it was used for the timeous identification of occult cerebral ischemia and hypoxia.

Some studies have shown that increased extracorporeal membrane oxygenation (ECMO) blood flow and sweep gas flow are effective at correcting low rSO2 [12]. However, the use of ECMO can cause complications and result in an economic burden. Conversely, IABP is easy to manage, is relatively cheap, has a wide range of indications (e.g., complications associated with acute myocardial infarction and pump failure), can reduce the load on the heart, and can increase organ perfusion[13]. In this case study, IABP was shown to be efficient.

Therefore, we recommend that the rSO2 of patients who are in a coma should be monitored routinely and continuously. An IABP may be an effective means of ensuring adequate brain oxygenation in patients who have experienced CA [14].

Further studies are needed to explore the application of cerebral oxygen monitoring.

## Conclusion

In this case study, cerebral oxygen monitoring, which was conducted throughout the treatment period after resuscitation, helped the doctors understand the patient’s cerebral perfusion in real time. This facilitated timely interventions, resulting in reduced occult brain injury and a good prognosis. The approach creates possible routes for treatment after resuscitation in patients who have experienced sudden death. Furthermore, hemodynamics should be optimized based on rSO2 levels to improve patients’ neurological prognoses. The limitation of this study is that it lacks sufficient data to reveal the characteristics and influencing factors of dynamic changes in cerebral oxygenation.

## Data Availability

The datasets used and/or analysed during the current study available from the corresponding author on reasonable request.

## Abbreviations

ROSC: Return of spontaneous circulation
CA: Cardiac arrest
IABP: Intra-Aortic Balloon Pump
rSO2: Regional cerebral oxygen saturation
PTCA: Coronary Artery Balloon Dilation
EF: Ejection fraction
CPC: Cerebral performance categories
